# Gender-based differences in platelet function and platelet reactivity to P2Y_12_ inhibitors

**DOI:** 10.1371/journal.pone.0225771

**Published:** 2019-11-27

**Authors:** Marco Ranucci, Tommaso Aloisio, Umberto Di Dedda, Lorenzo Menicanti, Carlo de Vincentiis, Ekaterina Baryshnikova, for the Surgical and Clinical Outcome REsearch (SCORE) group

**Affiliations:** 1 Department of Cardiothoracic, Vascular Anaesthesia and Intensive Care, IRCCS Policlinico San Donato, Milan, Italy; 2 Department of Cardiac Surgery, IRCCS Policlinico San Donato, Milan Italy; Royal College of Surgeons in Ireland, IRELAND

## Abstract

**Background:**

Gender influences platelet biology. Women have a larger platelet count, but gender-based differences in platelet function remain debated. We performed a study addressing gender-based differences in platelet function using point-of-care platelet function tests (PFT).

**Methods:**

The patient population consisted of 760 cardiac surgery patients where preoperative PFT (multiple-electrode aggregometry [MEA]) were available. Platelet count and function at the ADPtest and TRAPtest were compared in the overall population and separately in patients with or without residual effects of P2Y_12_ inhibitors.

**Results:**

Women had a significantly (P = 0.001) higher platelet count but a non-significantly higher platelet reactivity to ADP. In clopidogrel-treated patients, the platelets ADP reactivity was significantly (P = 0.031) higher in women, and platelet count was the main determinant of platelet hyper-reactivity. Within patients under full clopidogrel effects, women with a platelet count ≥ 200,000 cells/μL had a significantly (P = 0.023) higher rate of high-on-treatment platelet reactivity (HTPR, 45.5%) with respect to males with a platelet count < 200,000 cells/μL (11.9%), with a relative risk of 6.2 (95% confidence interval 1.4–29).

**Conclusions:**

Our findings confirm that women have a larger platelet count than men, and that this is associated to a trend towards a higher platelet reactivity. HTPR is largely represented in women with a high platelet count. This generates the hypothesis that women requiring P2Y_12_ inhibitors could potentially benefit from larger doses of drug or should be treated with anti-platelet agents with a low rate of HTPR.

## Introduction

Platelet biology is influenced by gender [1–3]. Platelet count is higher in women, and a number of studies have highlighted that platelet activation is enhanced [4–6], due to a greater expression of many surface receptors [7, 8].

Women are more susceptible to aspirin resistance, with a rate of major coronary events and ischemic stroke under aspirin treatment that is not different with respect to controls [9, 10].

In patients under double anti-platelet therapy (DAPT) the interaction between gender and efficacy was absent [11, 12] or only demonstrated a trend towards a lower efficacy in women [13].

Most of the studies investigating gender-dependent differences in platelet function were based on platelet count only, obsolete tests (bleeding time), light-transmission aggregometry, optical aggregometry, or sophisticated laboratory tests. However, the recent randomized controlled trials investigating the efficacy of different P2Y_12_ inhibitors used point-of-care (POC) platelet function tests (PFT) to address platelet reactivity and high on-treatment platelet reactivity (HTPR) [14–16]. Overall, among the phenotypical factors leading to HTPR in patients under clopidogrel, gender female is considered [17].

A large study addressing gender-based differences in platelet function and platelet reactivity to P2Y_12_ inhibitors based on the currently available POC PFT is presently lacking. The present study is a large retrospective analysis of platelet count and function in cardiac surgery patients before surgery, using a POC PFT, and aimed to determine gender-based differences.

## Materials and methods

### Study design

The present study is a post-hoc analysis of three previous (two retrospective in 2012–2013 and one prospective in 2016–2017) studies performed at our institution between 2010 and 2017 [18–20]. In these studies, patients scheduled for cardiac operations were investigated with standard laboratory tests and POC PFT before surgery. Data from these studies were utilized to investigate the hypothesis that gender-based differences exist in platelet reactivity in patients with or without DAPT. The three studies were approved by the local Ethics committee that waived the need for an informed consent for the two retrospective studies (Ethics Committee Melegnano, approval number 2702, 15/2/2012) and requested a written informed consent for the prospective study (Ethics Committee San Raffaele Hospital, approval number 137/INT/2016). Data from the three studies were pooled together and differences in platelet count and function between women and male were analyzed separately for patients free from the effects of P2Y_12_ inhibitors or under full/residual effects of these drugs.

This work was supported by the IRCCS Policlinico San Donato which is a Clinical Research Hospital recognized and funded by the Italian Ministry of Health.

### Patient population

The overall patient population included 760 adult cardiac surgery patients, of whom 440 were free from the effects of P2Y_12_ inhibitors (ticlopidine, clopidogrel, prasugrel or ticagrelor) and 320 were assessed within 6 days from P2Y_12_ inhibitors discontinuation.

The only exclusion criterion was the evidence of congenital platelet disease of any kind.

### Data collection and definitions

The following data were collected: demographics (age, gender, weight, height, and body mass index [BMI]); obesity (BMI > 30 kg/m^2^); heart function details; presence of coronary artery disease; co-morbidities; use of P2Y_12_ inhibitors (with type of drug); days of discontinuation of these drugs; type of cardiac surgery; serum creatinine level (mg/dL); bilirubin level (mg/dL); hematocrit (%). For patients under DAPT, the standard dose was used (ticlopidine 250 mg twice a day; clopidogrel 75 mg/day, prasugrel 10 mg/day reduced to 5 mg if weight < 60 kg; ticagrelor 90 mg twice a day).

Platelet function tests were performed at variable times before surgery, from 6 days to immediately before the induction of anesthesia, using a multiple-electrode aggregometry (MEA, Multiplate, Roche Diagnostics, Indianapolis, IN) and are explained in a dedicated section.

### Platelet function tests

An amount of 3 mL of blood was drawn from a venous line and collected in hirudin-coated tubes provided by the manufacturer. Platelet aggregation testing on the MEA analyzer was performed as follows. Briefly, 300 μL of whole blood were added to an equal amount of preheated saline solution and platelet aggregation was tested after specific activation with adenosine diphosphate (ADP, ADPtest, 6.5 μM final concentration). P2Y_12_ receptors are inhibited by direct ADP receptor inhibitors (clopidogrel, ticlopidine, prasugrel and ticagrelor). Platelet aggregation was electronically measured for 6 minutes and expressed as units of area under the curve plotted over time (U). Normal reference range for ADPtest is 57–113 (U). HTPR was defined according to a recent consensus paper [21] as a MEA ADPtest value > 46 U in patients under non-discontinued treatment with P2Y_12_ inhibitors. This cut-off value was increased to > 60 U for a discontinuation time within 12and 24 hours before the PFT.

All the test were performed by a dedicated biologist (E.B.) in the hemostasis/coagulation laboratory of our institution.

### Statistics

For the purpose of this study, the dependent variable were (i) the platelet reactivity at the MEA ADPtest and (ii) the HTPR rate. The main independent variable was gender. Other potential determinants of the dependent variables were those listed in data collection and definitions section. Among them, platelet count was considered an important potential confounder of MEA ADPtest, and was included in adjusted models for platelet reactivity and HTPR rate.

Continuous variables are presented as median and interquartile range after verification of a non-normal distribution at the Kolmogorov-Smirnov test, and as number and percentage for categorical variables. Differences between continuous variables were tested with non-parametric tests. Differences in categorical variables were tested using a Pearson’s chi square, producing relative risk and 95% confidence interval. Linear regression models were applied to adjust for potential confounders the relationship between gender and platelet function. Logistic regression models with dichotomous dependent variable were used to identify the independent variables and to adjust for potential confounders, producing odds ratio with 95% confidence interval. A P value < 0.05 was considered significant for all the statistical tests. All the analyses were performed using computerized statistical packages (SPSS 13.0, IBM, Chicago, IL, GraphPad Prism 7.0, GraphPad Software, Inc., La Jolla, CA)

## Results

The general characteristics and the platelet count and function data are presented in Table 1, separately for patients with or without residual effects of P2Y_12_ inhibitors. Apart from the normal anthropometric differences, within patients free from P2Y_12_ inhibitors women had a higher ejection fraction and NYHA class; a lower rate of stable coronary disease and diabetes; a lower hematocrit, serum creatinine value, and a higher serum bilirubin value. With respect to the platelet-related parameters, women had a significantly (P = 0.001) higher platelet count and a non-significantly (P = 0.085) higher platelet reactivity to ADP.

**Table 1:** General characteristics and platelet-related variables of the patient population (N = 760).

**Patients free from P2Y12 inhibitors (N = 440)**			
**Variable**	**Women (N = 146)**	**Men (N = 294)**	**P**
Age (years)	73 (61–78)	68 (59–74)	0.001
Weight (kgs)	62 (56–70)	78 (70–88)	0.001
Height (cms)	160 (156–165)	172 (168–178)	0.001
Body mass index	23.8 (21.4–27.3)	26 (23.9–29.3)	0.001
Obesity	24 (16.4)	61 (20.7)	0.281
Ejection fraction (%)	55 (50–62)	55 (47–60)	0.019
New York Heart Association class	0 (0–2)	0 (0–2)	0.005
Stable coronary artery disease	42 (28.8)	149 (50.7)	0.001
Unstable angina	0 (0)	0 (0)	N/A
Congestive heart failure	14 (9.6)	19 (6.5)	0.241
Chronic obstructive pulmonary disease	12 (8.2)	18 (6.1)	0.411
Previous cerebrovascular accident	6 (4.1)	9 (3.1)	0.568
Diabetes on medication	17 (11.6)	58 (19.7)	0.034
Serum creatinine (mg/dL)	1.0 (0.8–1.4)	1.2 (1.0–1.4)	0.001
Serum bilirubin (mg/dL)	0.5 (0.5–0.6)	0.5 (0.5–0.5)	0.039
Hematocrit (%)	38 (34–40)	42 (39–44)	0.001
Platelet count (x 1,000 cells/μL)	211 (173–266)	191 (158–225)	0.001
Platelet reactivity at ADP test (U)	58 (45–74)	56 (39–70)	0.085
**Patients under or with residual effects of P2Y12 inhibitors (N = 320)**			
**Variable**	**Women (N = 64)**	**Men (N = 256)**	**P**
Age (years)	75 (66–79)	67 (60–74)	0.001
Weight (kgs)	65 (55–76)	77 (70–83)	0.001
Height (cms)	160 (155–165)	170 (166–175)	0.001
Body mass index	24.8 (22–31)	26.4 (24.2–28.4)	0.124
Obesity	17 (26.6)	34 (13.3)	0.009
Ejection fraction (%)	50 (42–57)	53 (45–60)	0.148
New York Heart Association class	0 (0–2)	0 (0–1)	0.982
Stable coronary artery disease	39 (60.9)	188 (73.4)	0.049
Unstable angina	6 (10.9)	14 (5.9)	0.183
Congestive heart failure	4 (7.3)	18 (7.6)	0.941
Chronic obstructive pulmonary disease	5 (9.1)	19 (8)	0.787
Previous cerebrovascular accident	0 (0)	4 (1.7)	0.333
Diabetes on medication	11 (20)	58 (24.4)	0.491
Serum creatinine (mg/dL)	0.9 (0.7–1.3)	1.0 (0.8–1.2)	0.233
Serum bilirubin (mg/dL)	0.5 (0.3–0.7)	0.55 (0.3–0.7)	0.135
Hematocrit (%)	37 (34–39)	39.4 (35.6–41.6)	0.001
Platelet count (x 1,000 cells/μL)	220 (173–268)	189 (158–226)	0.001
Platelet reactivity at ADP test (U)	41 (22–63)	32 (20–53)	0.148
Platelet reactivity at ADP test (U)°	46 (26–65)	34 (22–56)	0.031
Days of discontinuation	2 (1–4.7)	2 (1–4)	0.712
P2Y_12_ inhibitor			0.442
Ticlopidine	7 (10.9)	20 (7.8)	
Clopidogrel	52 (81.3)	216 (84.7)	
Prasugrel	1 (1.6)	10 (3.9)	
Ticagrelor	4 (6.3)	9 (3.5)	
High on treatment platelet reactivity°°	7 (35)	15 (18.1)	0.097

Data are median (interquartile range) or number (%). ADP: adenosindiphosphate

° Clopidogrel-treated patients only

°°No discontinuation or discontinuation time ≤ 24 hours before the test (N = 103, 20 women and 83 men).

Once adjusted for the platelet count, the ADP-dependent platelet reactivity lost even this non-significant trend for association with gender (regression coefficient 0.594, 95% confidence interval -3.87 to 4.96, P = 0.789), while platelet count was a significant determinant of platelet reactivity (regression coefficient 0.164, 95% confidence interval 0.132–0.195, P = 0.001).

Within patients under full or residual effects of P2Y_12_ inhibitors, women demonstrated a larger obesity rate, a lower rate of stable coronary disease and a lower hematocrit value.

The higher platelet count in women was confirmed within this subgroup. Platelets ADP reactivity was non-significantly (P = 0.148) higher in women, and the rate of HTPR was similar in women and men. When the analysis was restricted to clopidogrel-treated patients, the platelets ADP reactivity was significantly (P = 0.031) higher in women.

**Fig 1:**
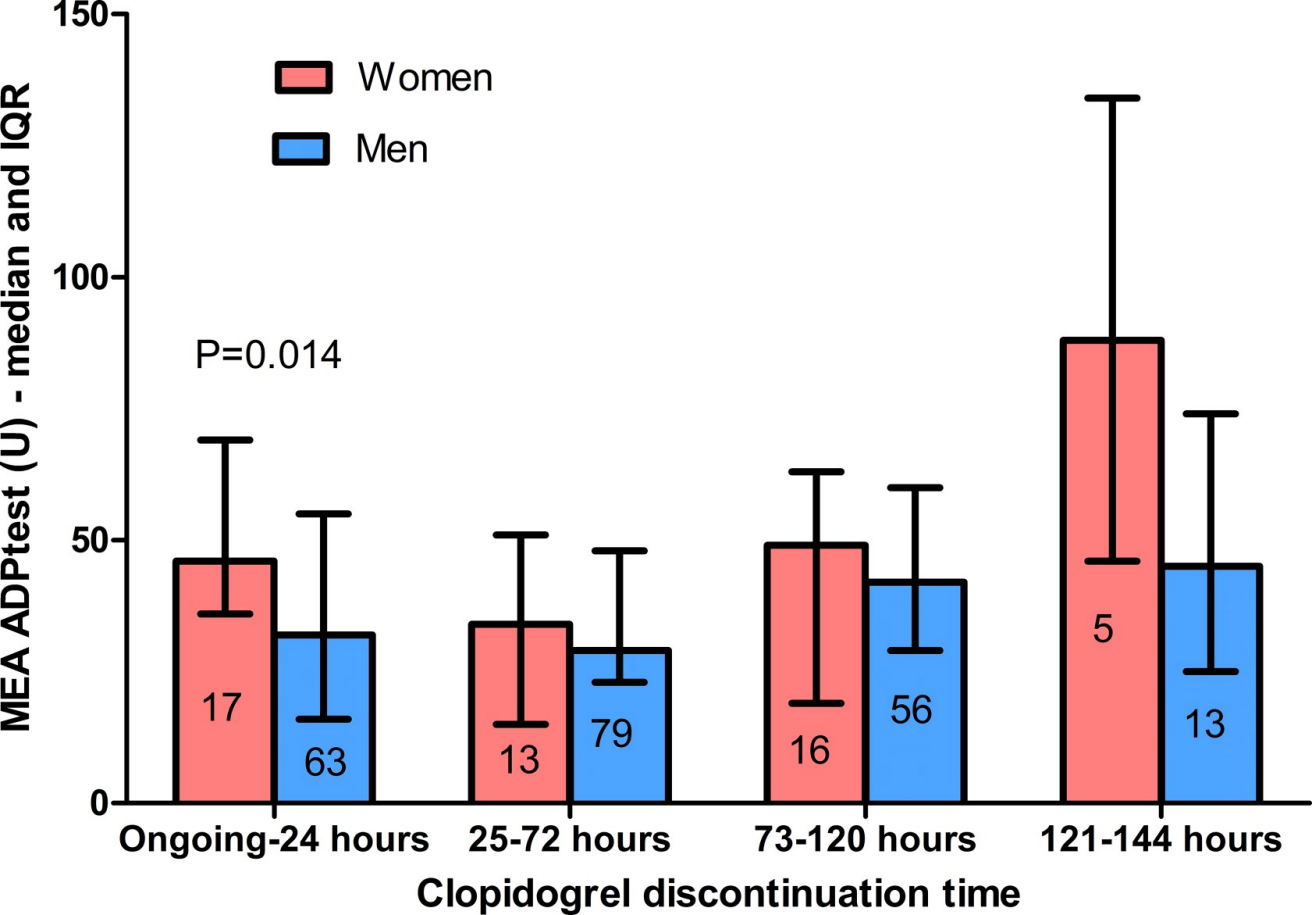
Platelet reactivity at the adenosine diphosphate (ADP) test in women and men, depending on the clopidogrel discontinuation time. IQR: interquartile range; MEA: multiple electrode aggregometry. Numbers within boxes are the number of subjects. The independent variables associated with HTPR are presented in Table 2.

The values of ADP reactivity test as a function of the time of discontinuation are shown in Fig 1. This analysis is restricted to patients receiving clopidogrel within 6 days before the PFT. Within patients on full clopidogrel treatment or who discontinued clopidogrel within 24 hours before the PFT, the ADP-dependent reactivity of platelets was significantly (P = 0.014) higher in women (median 46 U, interquartile range 36–69) than in men (median 32 U, interquartile range 16–55). Once corrected for platelet count, gender female demonstrated a non-significant (P = 0.061) trend towards a higher platelet reactivity, while platelet count was significantly (P = 0.008) associated with a higher platelet reactivity. In this subgroup, there was a non-significant (P = 0.097) higher rate of HTPR in women (relative risk 2.44, 95% confidence interval 0.83–7.16).

**Table 2:** Factors associated with high on-treatment platelet reactivity (N = 103).

Item	HTPR (N = 22)	No HTPR (N = 81)	Relative risk (95% C.I.)	P
Age (years)	69 (62–77)	68 (60–75)	N/A	0.435
Gender female	7 (31.8)	13 (16)	2.44 (0.83–7.16)	0.097
Weight (kgs)	76 (69–85)	75 (69–83)	N/A	0.832
Height (cms)	160 (160–171)	170 (165–175)	N/A	0.017
Body mass index	27.5 (23.7–31)	26.0 (23.8–28.1)	N/A	0.288
Obesity	6 (27.3)	11 (13.6)	2.38 (0.77–7.41)	0.125
Ejection fraction (%)	51 (40–57)	53 (45–60)	N/A	0.674
New York Heart Association class	0 (0–0)	0 (0–0)	N/A	0.551
Stable coronary artery disease	14 (63.6)	49 (60.5)	1.14 (0.43–3.03)	0.789
Unstable angina	4 (21.1)	4 (5.4)	4.67 (1.05–20.8)	0.030
Congestive heart failure	1 (5.3)	5 (6.8)	0.77 (0.08–6.98)	0.813
Chronic obstructive pulmonary disease	5 (26.3)	3 (4.1)	8.45 (1.81–39.5)	0.002
Previous cerebrovascular accident	0 (0)	1 (1.4)	N/A	0.610
Diabetes on medication	5 (26.3)	13 (17.6)	1.68 (0.51–5.47)	0.389
Serum creatinine (mg/dL)	0.9 (0.8–1.1)	1.0 (0.8–1.1)	N/A	0.711
Serum bilirubin (mg/dL)	0.6 (0.4–0.6)	0.6 (0.5–0.7)	N/A	0.367
Hematocrit (%)	38.5 (32–40)	37.6 (34.3-4o)	N/A	0.454
Platelet count (x 1,000 cells/μL)	234 (186–280)	193 (154–235)	N/A	0.027

Data are median (interquartile range) or number (%). N/A: not applicable.

Factors with a P value < 0.1 were included in a multivariable analysis: for this purpose, continuous variables were dichotomized according to the median value. Within this model, factors independently associated with HTPR were unstable angina (odds ratio 6.0, 95% confidence interval 1.16–31, P = 0.033) and chronic obstructive pulmonary disease (odds ratio 7.2, 95% confidence interval 1.42–36, P = 0.017). Gender female was not independently associated with HTPR (odds ratio 2.14, 95% confidence interval 0.58–7.91, P = 0.253). A second model inclusive of gender female and platelet count only yielded an odds ratio for HTPR of 2.40 (95% confidence interval 0.80–7.20, P = 0.118).

**Fig 2:**
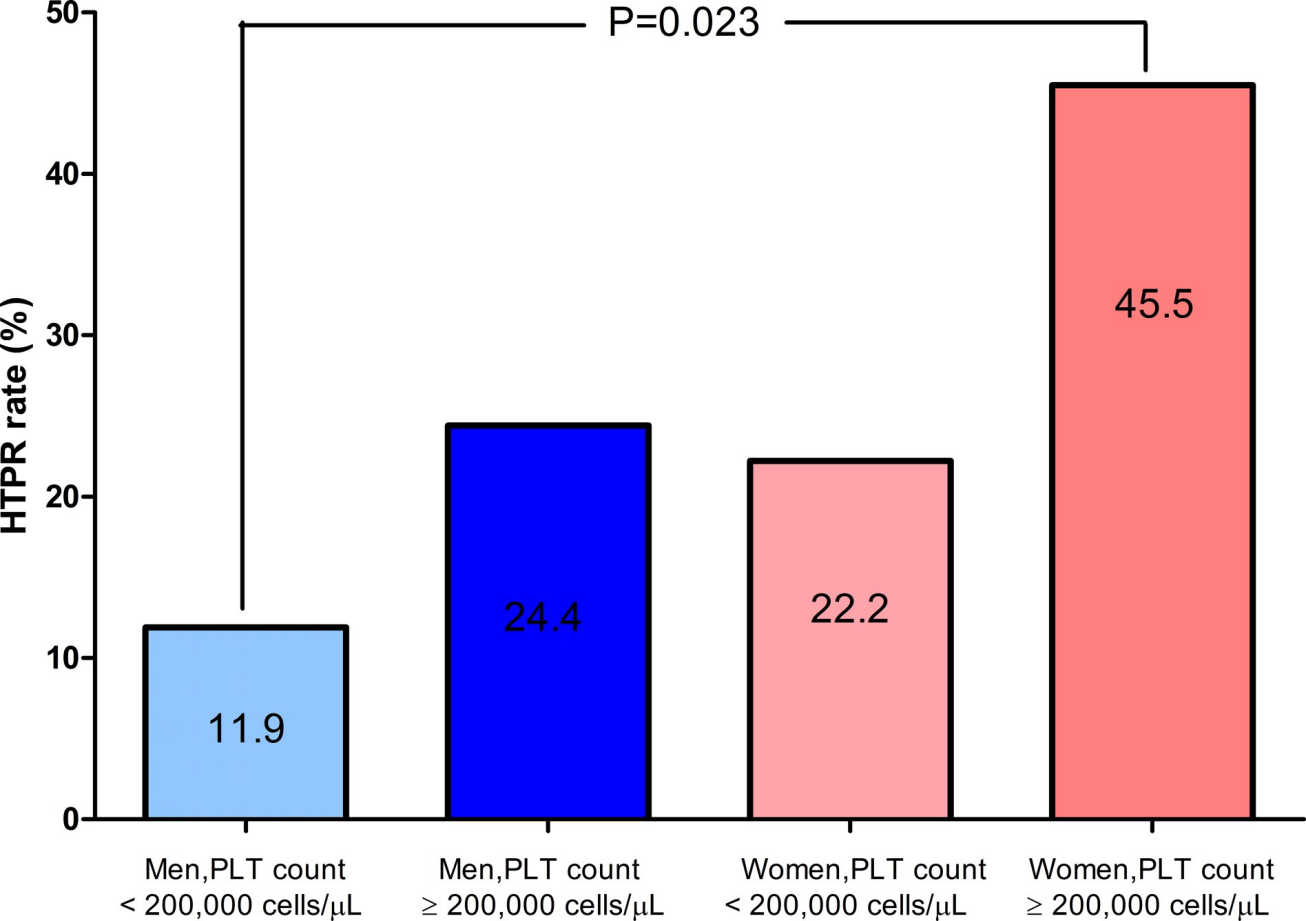
High-on-treatment platelet reactivity (HTPR) in women and men under full clopidogrel effects, as a function of platelet count. PLT: platelet.

The patient population was divided into four classes depending on gender and platelet count below or above the value of 200,000 cells/μL, that corresponds to the median value in the overall population (Fig 2). Female patients with a platelet count ≥ 200,000 cells/μL had the higher rate of HTPR (45.5%) with a significant (P = 0.023) difference with respect to males with a platelet count < 200,000 cells/μL (11.9%), with a relative risk of 6.2 (95% confidence interval 1.4–29).

## Discussion

The main results of the present study may be summarized as follows: (i) in patients free from the effects of P2Y_12_ inhibitors, gender female is associated with a higher platelet count but not with a higher ADP-dependent platelet reactivity; (ii) in patients under full or residual effects of P2Y_12_ inhibitors of any kind, the ADP-dependent platelet reactivity was not significantly different between women and men; and (iii) in patients under full or residual effects of clopidogrel, the ADP-dependent platelet reactivity was significantly higher in women, especially in those who discontinued the drug within 24 hours before the test; however, only a trend remained after correction for platelet count; (iv) in patients who discontinued the P2Y_12_ inhibitors within 24 hours before the PFT, after correction for the potential confounders, the risk of HTPR was double in women vs. men, however without reaching a statistical significance. A combination of gender female and platelet count ≥ 200,000 cells/μL yielded a high risk of HTPR (45%).

Overall, our study seems to confirm a general trend towards a higher ADP-dependent platelet reactivity in women treated with P2Y_12_ inhibitors and namely with clopidogrel, however raising the possibility that this finding is dependent on differences in platelet count rather than function. Multiple-electrode aggregometry is based on the ability of platelets to aggregate on electrical filaments and hence to increase the electrical impedance. The increase in electrical impedance is not only dependent on the quality of platelet aggregation, but even on the quantity of platelets involved in the process. Previous studies have highlighted that the results of MEA have a positive association with platelet count, regardless of platelet count value [22]. A high platelet count has been already identified as a factor leading to HTPR [17, 18]. Other point-of-care tests of platelet function may have different associations with platelet count, but it reasonable to hypothesize that thrombocytosis affects platelet function measurements.

Even considering the confounding effect of higher platelet counts, a signal towards a higher rate of HTPR in women remains: after correction for platelet count, the odds ratio for HTPR is more than double in females, lacking statistical significance due to the relatively low sample size (103 patients). The combination of gender female and a high platelet count carries a risk for HTPR that is largely higher than what reported in the existing Literature [17,18, 21].

Data on the role of gender in the determinism of HTPR are presently scarce. A cohort study on 846 treated with prasugrel or clopidogrel identified gender as a covariate affecting platelet inhibition in a linear mixed-effect model comparing the maximum platelet aggregation and the inhibition of platelet aggregation between treatments over time points [23]. A recent Danish study on stroke patients treated with clopidogrel reports that patients with HTPR were 46% females vs. 39% in non-HTPR patients [24]. This difference is larger in a recent study where gender female was found in 29% of HTPR patients vs. 15% in non-HTPR patients [25].

P2Y_12_ inhibitors commonly used for DAPT belong to thienopyridines (ticlopidine, clopidogrel, prasugrel) which irreversibly block the ADP receptor), or cyclopentyl triazolopyrimidines, which exert a reversible inhibition (ticagrelor). Cilostazol is not used in coronary patients. HTPR has been described in about 25% of patients treated with clopidogrel [18], 10% of patients treated with prasugrel, and 2% in patients treated with ticagrelor [26]. Other differences among these drugs include a more rapid onset of action for prasugrel and ticagrelor vs. clopidogrel [27].

Studies investigating the effects of prasugrel and ticagrelor failed to detect gender-related differences in outcomes [11,12]. In these studies, the prevalence of gender female was between 25% and 28%. However, even if no differences in outcomes have been demonstrated, there are evidences that coronary patients under DAPT have different gender-based platelet reactivity and HTPR rate. In a recent study, Jastrszebka and associates found that women treated with a combination of aspirin and clopidogrel had a higher residual platelet reactivity than men, and a higher HTPR [28].

The physiological basis for a higher platelet reactivity in women, apart from their larger platelet count, has been hypothesized in previous studies. Platelets have estrogen receptors that inhibits platelet activation in pre-menopausal women [29]. This effect is lost in post-menopausal women (the large majority of our patient population). Additionally, estrogens are involved in prostacyclines synthesis and nitric oxide bioavalability [30], both factors blunting platelet aggregation. In pre-menopausal women, estrogen levels fluctuations may result in different levels of platelet reactivity.

As clopidogrel has to be metabolized by cytochrome P (CYP) enzymes, it is possible that there is an estrogen-dependent effect on CYP activity. Actually, there is an estrogen-dependent effect on cytochrome P450 3A (CYP3A) subfamily: in the endometrium, CYP3A4 and CYP3A43 are down-regulated by estrogen [31]. However, polymorphisms of CYP3A4 were not associated with treatment resistance when compared to responders to clopidogrel therapy [32].

In a study population similar to our study (coronary patients with stable angina) [28], the authors could find that in gender female the higher platelet reactivity while on DAPT with clopidogrel was associated with a pro-inflammatory profile, with higher levels of C-reactive protein and leukocytes.

There are limitations in our study. The first is the retrospective nature which did not allow us to address some possible confounders, like smoking habit. The second is the relatively low sample size with consequent no-significance of some potentially relevant trends. Finally, the great majority of our patients was treated with clopidogrel, which is a drug with a considerable HTPR rate. It is possible that the results differ if tested in patients under P2Y_12_ inhibitors with a lower rate of HTPR (prasugrel or tcagrelor).

Given these limitations, our study highlights that the subset of women with a high platelet count seems particularly susceptible to HTPR, at a rate (45%) larger than the expected (25% from the existing literature). This finding generates the hypothesis that this segment of patients, when requiring P2Y_12_ inhibitors, could potentially benefit from larger doses or, more reasonably, be directly treated with drugs carrying the lower risk of HTPR (ticagrelor). Association with oral anti-factor Xa drugs could be an additional option in patients requiring a prophylaxis of thrombotic events (atrial fibrillation). Further studies in these directions are warranted.

## Supporting information

S1 Table Original data. (XLS)
